# Spatial analysis of lung, colorectal, and breast cancer on Cape Cod: An application of generalized additive models to case-control data

**DOI:** 10.1186/1476-069X-4-11

**Published:** 2005-06-14

**Authors:** Verónica Vieira, Thomas Webster, Janice Weinberg, Ann Aschengrau, David Ozonoff

**Affiliations:** 1 Department of Environmental Health, Boston University School of Public Health, 715 Albany Street, Boston, MA 02118, USA; 2 Department of Biostatistics, Boston University School of Public Health, 715 Albany Street, Boston, MA 02118, USA; 3 Department of Epidemiology, Boston University School of Public Health, 715 Albany Street, Boston, MA 02118, USA

## Abstract

**Background:**

The availability of geographic information from cancer and birth defect registries has increased public demands for investigation of perceived disease clusters. Many neighborhood-level cluster investigations are methodologically problematic, while maps made from registry data often ignore latency and many known risk factors. Population-based case-control and cohort studies provide a stronger foundation for spatial epidemiology because potential confounders and disease latency can be addressed.

**Methods:**

We investigated the association between residence and colorectal, lung, and breast cancer on upper Cape Cod, Massachusetts (USA) using extensive data on covariates and residential history from two case-control studies for 1983–1993. We generated maps using generalized additive models, smoothing on longitude and latitude while adjusting for covariates. The resulting continuous surface estimates disease rates relative to the whole study area. We used permutation tests to examine the overall importance of location in the model and identify areas of increased and decreased risk.

**Results:**

Maps of colorectal cancer were relatively flat. Assuming 15 years of latency, lung cancer was significantly elevated just northeast of the Massachusetts Military Reservation, although the result did not hold when we restricted to residences of longest duration. Earlier non-spatial epidemiology had found a weak association between lung cancer and proximity to gun and mortar positions on the reservation. Breast cancer hot spots tended to increase in magnitude as we increased latency and adjusted for covariates, indicating that confounders were partly hiding these areas. Significant breast cancer hot spots were located near known groundwater plumes and the Massachusetts Military Reservation.

**Discussion:**

Spatial epidemiology of population-based case-control studies addresses many methodological criticisms of cluster studies and generates new exposure hypotheses. Our results provide evidence for spatial clustering of breast cancer on upper Cape Cod. The analysis suggests further investigation of the potential association between breast cancer and pollution plumes based on detailed exposure modeling.

## Background

Local disease mapping ("cluster") investigations are often desired by concerned communities, but many epidemiologists resist the pressure to search for environmental causes of clusters. Critics argue that such studies are unproductive and flawed because they often combine unrelated diseases, apply arbitrary or even "gerrymandered" boundaries, contain insufficient numbers of cases, and ignore population density, latency, and known risk factors [1]. Data based on cancer registries are generally mapped by town of diagnosis (or other geographic unit) and contain limited data on covariates. This results in poor spatial resolution, potential spatial confounding, and the inability to consider latency. Spatial confounding occurs when risk factors for a disease are not evenly distributed, e.g., a cluster of lung cancer may be due to an increased density of smokers. Since cancer typically takes many years to develop, residence at diagnosis is likely to be a poor measure of exposure. Maps that ignore latency may tend to be flatter if population movement is random with respect to disease status [2]. Nevertheless, cluster investigations can be an important part of responding to public concerns, even if no new etiologic knowledge is gained [3,4].

**Figure 1:**
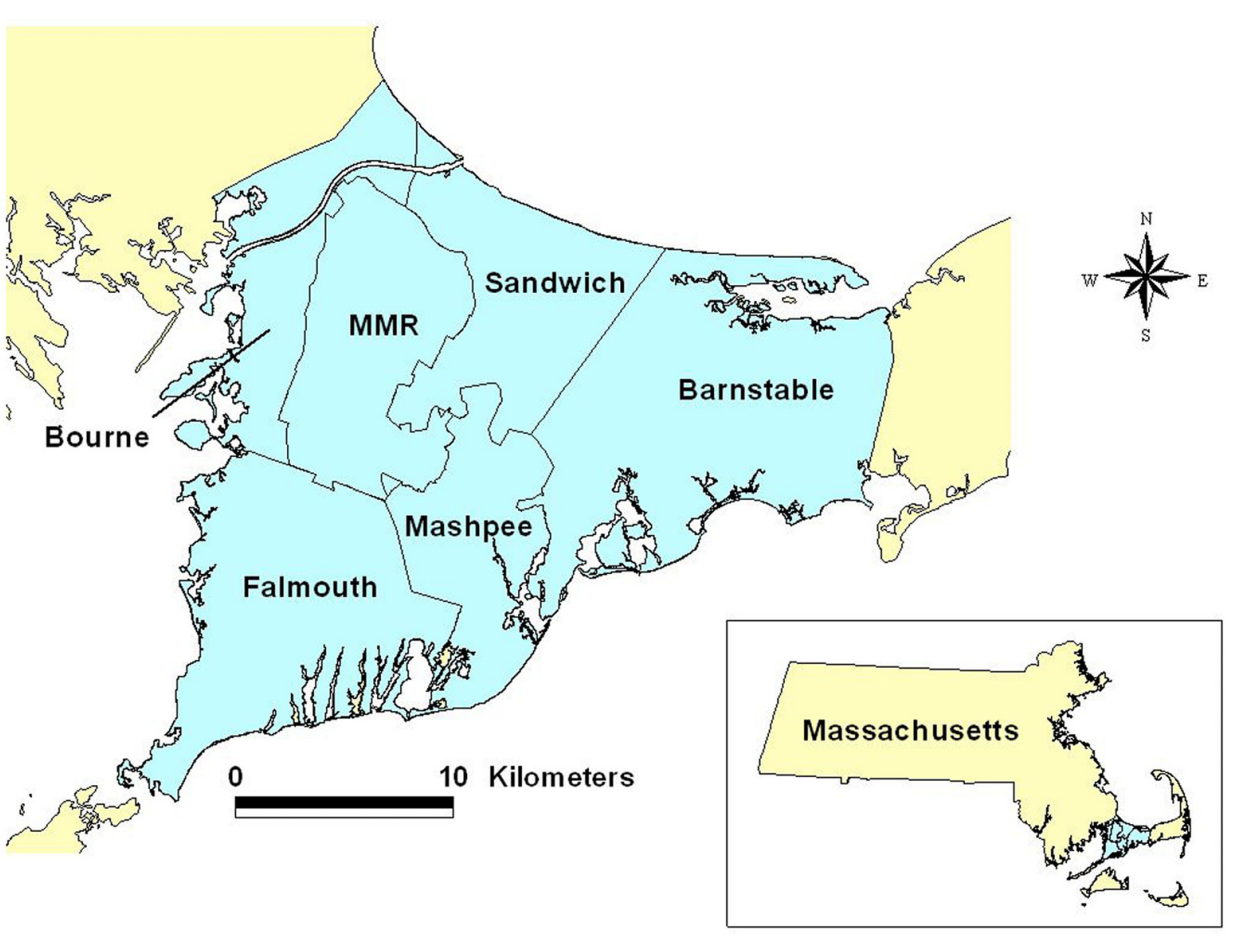
**Geographic location of the upper Cape Cod study area. **Cape Cod is located in Massachusetts in the northeast United States.

In 1988, an elevated cancer incidence in the Upper Cape Cod region of Massachusetts (Figure 1) prompted a series of epidemiological studies to investigate possible environmental risk factors, including air and water pollution associated with the Massachusetts Military Reservation (MMR), pesticide applications to cranberry bogs, particulate air pollution from a large electric power plant, and tetrachloroethylene-contaminated drinking water from vinyl-lined asbestos cement distribution pipes [5-15]. Positive associations were observed, but the environmental exposures explained only a portion of the excess cancer incidence. These studies provide an invaluable data set for spatial analysis. Population-based case-control studies can provide detailed information on individual-level covariates and residential history. Cases are identified using cancer registries while controls provide an estimate of the underlying population density. Subjects or next-of-kin are interviewed to obtain relevant data on covariates and residential history. Geocoding of this information produces a rich, point-based data set that can be analyzed with the help of geographical information systems (GIS).

Methods for mapping point-based epidemiologic data have received less attention than mapping areal data [16]. Generalized additive models (GAMs), a type of statistical model that combines smoothing with the ability to analyze binary outcome data and adjust for covariates, provide a useful framework for examining such point data [17-19], Webster et al. submitted. Using individual-level information and location in a generalized additive model, we calculated the crude and adjusted odds ratios for lung, colorectal, and breast cancers on Upper Cape Cod assuming different latency periods. These analyses have several objectives: i) to test if the disease maps are flat, ii) to determine if areas of increased or decreased risk are due to spatial confounding, iii) to examine the effect on the maps of increasing latency, iv) to suggest exposure hypotheses for further investigation, and v) to demonstrate spatial epidemiology using generalized additive models.

## Methods

### Study Population

We investigated the association between residence and breast, lung and colorectal cancer on Upper Cape Cod, Massachusetts (USA) using data from population-based case-control studies [10-12]. The Massachusetts Cancer Registry was used to identify incident breast cancer cases diagnosed from 1983–1993 and incident cases of lung and colorectal cancers diagnosed from 1983–1986. Participants were restricted to permanent residents of the upper Cape region with complete residential histories. A total of 638 breast cancer cases, 243 lung cancer cases, and 309 colorectal cancer cases were included.

Controls were chosen to represent the underlying population that gave rise to the cases, i.e., permanent residents of the same towns during the same time period. Controls were frequency matched to cases on age, gender, and vital status. Because many of the cases were deceased or elderly, three different sources of controls were used: (1) random digit dialing for living controls less than 65 years of age; (2) Centers for Medicare and Medicaid Services (formerly the Health Care Financing Administration) for the living population 65 years of age or older; and (3) death certificates for controls who had died from 1983 onward. There were 842 breast cancer controls, 1205 lung cancer controls, and 1138 colorectal cancer controls.

Participants or their next-of-kin completed an extensive interview, providing information on demographics (age, sex, marital status, and education), a forty-year residential history, and potential confounders. "Index years" were randomly assigned to controls in a distribution similar to that of diagnosis years for cases. We used index years to estimate length and time of environmental exposure for controls in a fashion comparable to that of cases. See earlier papers [10-12] for a detailed description of the methods used to define the study population, including the rationale for the method of control selection. The Institutional Review Board of Boston University Medical Center approved the research.

### Geographical Information System (GIS)

All residential addresses reported by participants in the upper Cape Cod area over the forty-year period prior to the diagnosis or index year were eligible for spatial analysis. We excluded all addresses where residency time began after diagnosis date for cases or index date for controls. The breast cancer data set included 638 cases representing 1061 residential locations and 842 controls representing 1371 locations. The lung cancer data set included 243 cases representing 385 residential locations and 1205 controls representing 1927 residential locations. The colorectal cancer data set included 309 cases representing 469 residential locations and 1138 controls representing 1791 residential locations. Thus, individual participants may have contributed more than one address.

**Figure 2:**
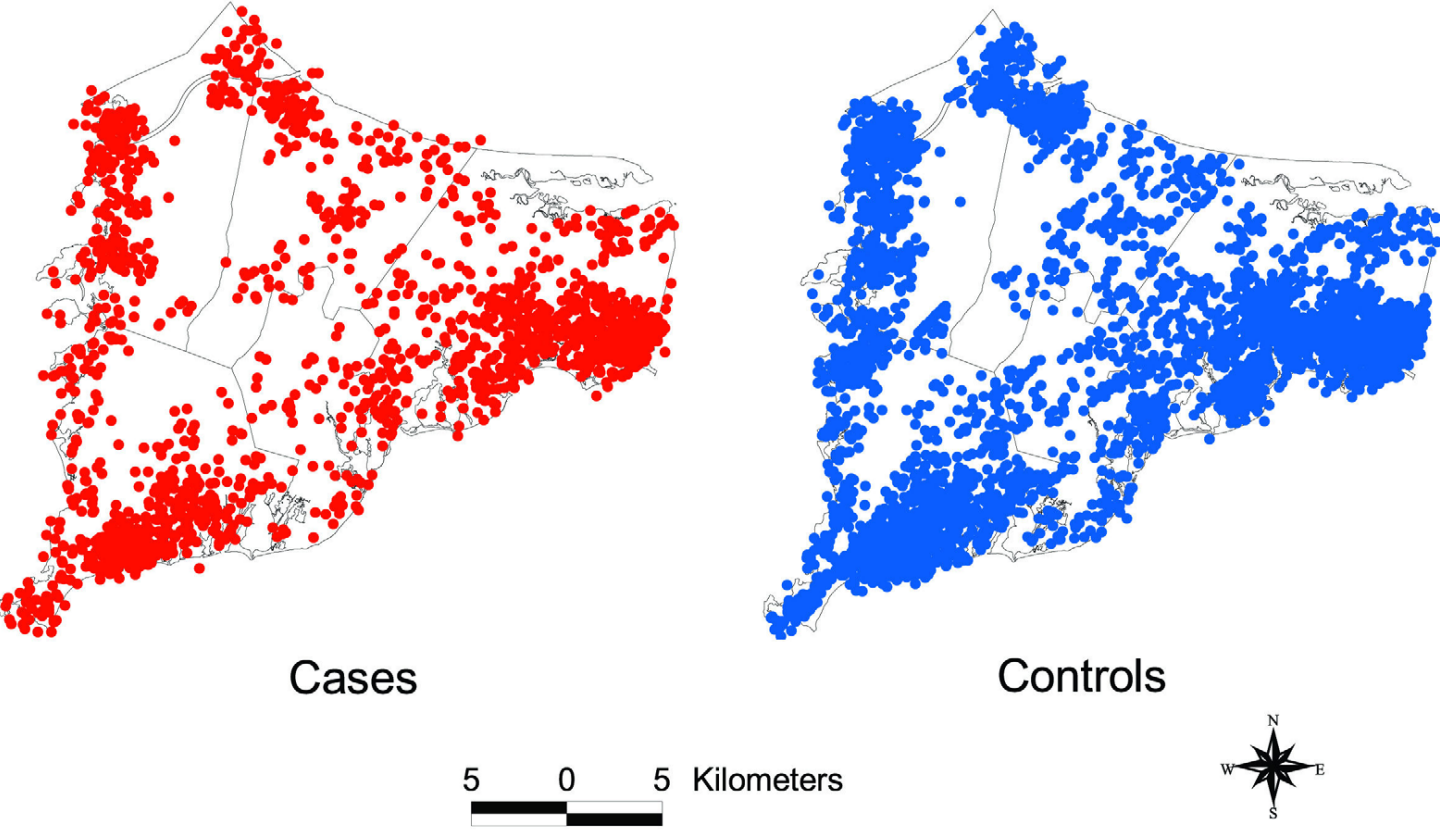
**Distribution of cases and controls for lung, colorectal, and breast cancer. **Each point represents the residence of one participant. Locations have been geographically altered to preserve confidentiality.

Locations of the participant residences were geocoded using the Massachusetts State Plane Coordinate System with North American Datum 1983 (NAD1983) and linked to the participant's interview data. Geocoding, the process where longitude and latitude are determined for each street address, was done without knowledge of case status, and the final data were checked for accuracy. GIS allows us to map the coordinates of participants and link the location to additional individual and environmental information. Figure 2 shows the distribution of lung, colorectal, and breast cancer cases and controls in the study area. To preserve confidentiality, the figure was created by randomly placing residences within a small grid that includes the actual location. Actual locations were used in the analysis.

### Statistical methods for mapping point-based epidemiologic data

Statistical methods for mapping area-based epidemiologic data, e.g., disease rates by town or county, are well advanced [20]. Mapping such data often has two main components: adjusting for covariates, often via standardization, and contending with geographically varying degrees of precision, often by smoothing. Methods for point-based data are less well developed [16]. One approach uses kernel methods to estimate the density of cases and the density of the population giving rise to the cases [21,22]. Their ratio provides an estimate of the rate. Alternatively, one estimates the density of the population using controls. When controls are appropriately sampled from the population of a geographic area, the case/control ratio – disease odds – in a sub-area should be proportional to the disease incidence rate in that sub-area. Unfortunately, the density ratio approach provides no easy method to adjust for covariates [23]. Other multi-step methods have been suggested [24,25].

At least two methods provide unified frameworks for mapping point-based epidemiologic data, adjusting for covariates, and hypothesis testing: generalized linear mixed model formulations of kriging [15,23,26,27] and generalized additive models (GAMs) using bivariate kernel or loess smoothers [18,19,23]. Both are promising but relatively untried methods in spatial epidemiology. For example, kernel-based GAMs have been used to map risks of lung cancer [18], biliary cirrhosis [28], and infant mortality [29].

### Mapping via generalized additive models (GAMs)

We estimated local disease odds using generalized additive models, a form of non-parametric or semi-parametric regression with the ability to analyze binary outcome data while adjusting for covariates [17]. We modeled location, a potential proxy measure of exposure, using a bivariate smooth (S) of latitude (x_1_) and longitude (x_2_)

          logit [p(x_1_,x_2_)] = S(x_1_,x_2_) + γ '**z **    (1)

where the left-hand side is the log of the disease odds at location (x_1_,x_2_), **z **is a vector of covariates, and γ is a vector of parameters. The model is semiparametric because it has both nonparametric and parametric components. Without the smooth function, S(x_1_,x_2_), the model becomes an ordinary logistic regression on the covariates. Omitting the covariates produces a crude (unadjusted) map. We used a loess smooth which adapts to changes in population density [17]. The amount of smoothing depends on the percentage of the data points in the neighborhood, referred to as the span size. GAMs also allow selection of "optimal" span size and hypothesis testing. Webster et al. (submitted) provides a detailed discussion of the statistical methods, analyses using synthetic data, and a comparison with the kernel method of Kelsall and Diggle [18]. We used S-Plus [30] to perform the generalized additive modeling and ArcView [31] to map the results of our analyses. Program code is available on request.

We determined the optimal amount of smoothing for each map by minimizing the Akaike's Information Criterion (AIC). Small span sizes produce bumpier surfaces and larger span sizes produce smoother surfaces. As the span size increases, the amount of bias in the fit increases and the variance decreases [17]. We created a rectangular grid covering the study area using the minimum and maximum latitude and longitude coordinates from the original data set. Grid points lying outside the outline map of the study area were clipped, as were areas where people cannot live (e.g., ocean or wildlife refuges). We estimate the crude and adjusted log odds at each location on the grid using the S-Plus function *predict.gam*. As this function defines neighborhoods based on a combination of the data points and the grid, it can produce discrepancies from predictions based on the original data alone [32,33]. We therefore checked all maps and found that any discrepancies were minor, not changing our conclusions.

We converted from log odds to odds ratios (ORs) using the whole study area as the reference, dividing the odds at each grid point by the odds calculated by the reduced model omitting the location smoothing term. The odds ratio estimates the rate ratio and relative risk. In order to make maps visually comparable, we mapped all results using the same dark blue to dark red continuous (unclassified) color scale and range of odds ratios, 0.25–2.50. This range covers most but not all of the ORs observed in our analyses, preventing maps from being washed out by an area of extremely high or low ORs. We used a linear scale for odds ratios; although a log scale is a good option, it may be more difficult for many people to interpret. As odds ratios near unity appear as a light green, this scale is close to divergent, an effective way to communicate deviations of the map from flatness in both directions. Spectral scales, such as ours, are useful when there is a clear central value – here, an odds ratio of one – from which divergence is important (See Brewer et al [34] for a useful discussion of color schemes). Cancer maps have historically used blue and red for areas of low and high rates [35]. Blue and red are commonly associated with cold and hot, aiding interpretability of areas with decreased or increased risk.

We determined the presence of spatial confounding by visually comparing crude and adjusted maps. If their optimal span sizes differ, we also compared maps using a common span, allowing us to distinguish between changes due to adjustment and changes due to span.

GAMs also provide a framework for testing hypotheses. There are a number of ways to test the global null hypothesis that disease status does not depend on location, i.e., that the map is flat. Similar to analysis of variance in ordinary linear regression, we examined the overall significance of location using the difference in deviance of the complete model (equation 1) and the reduced model omitting the smoothing term. The S-Plus software provides an approximate p-value for this statistic assuming a chi square distribution. Because the latter assumption is in general not true for GAMs [17], we calculated the p-value using a permutation test. To test the null hypothesis of no association between case/control status and location, we randomly reassigned individuals to the eligible residences. This relabeling procedure preserves the number of cases and controls and the relationship between case/control status and covariates, but any deviation from a flat map is due to chance. We sampled from the null permutation distribution 999 times in addition to the original. For each permutation, we ran the GAM using the optimal span of the original data and computed the deviance statistic. We divided the rank of the observed value by 1000 to obtain the approximate permutation p-value. For comparison, we also computed a permutation p-value for the global statistic used by Kelsall and Diggle [18].

If the deviance global statistic indicated that location was significant at the 0.05 level, we conducted pointwise permutation tests to identify areas with significantly increased or decreased risk. We obtained a distribution of the log odds at every point using the same set of permutations we used for calculating the global statistics. The areas of significantly decreased risk ("cold spots") include all points that rank in the lower 2.5% of the pointwise distributions. Areas of significantly elevated risk ("hot spots") include all points that rank in the upper 2.5% of the pointwise distributions. By drawing the 2.5% and 97.5% contour lines, we mapped areas of significantly decreased and increased risk.

### Covariates and Missing Data

A group of core confounders, chosen *a priori *based on the current scientific literature or study design, was included in all adjusted analyses of breast cancer: time period of case ascertainment and vital status at interview, age at diagnosis or index year, family history of breast cancer, personal history of breast cancer (before current diagnosis or index year), age at first live birth or stillbirth, and occupational exposure to solvents. A number of other covariates were retained because they changed the appearance of the map: history of benign breast cancer, race, body mass index, history of radiation exposure, and alcohol use. We dropped other covariates from the model because they did not change the appearance of the map, including past use of diethylstilbestrol (DES), oral contraceptives and menopausal hormones, history of cigarette smoking, marital status, religion, education level, exposure to tetrachloroethylene from distribution pipes, and physical activity level. Lung cancer data were adjusted for age at diagnosis or index year, sex, vital status at interview, smoking (cigarettes, pipe, and cigars), living with a smoker, occupational exposure to lung carcinogens (jobs with arsenic, asbestos, chromium, coal tar pitch exposure), and exposure to radiation. Dropped covariates included alcohol history, use of pesticides/herbicides in the garden, exposure to tetrachloroethylene from distribution pipes, and whether the residence had been treated for termites. For colorectal cancer, we adjusted for age at diagnosis or index year, sex, vital status at interview, history of inflammatory bowel disease, and occupational history associated with colorectal cancer (jobs with asbestos or solvent exposure). History of alcohol use and radiation exposure did not affect the appearance of the maps.

We restricted analysis to subjects with complete residential histories. In our initial analyses, subjects missing data for other covariates were included in the analyses but those variables were coded as missing using an indicator variable [36]. While this method is often adequate in our experience, it can theoretically lead to bias [37]. Therefore, to ensure that positive results were not biased by the use of the indicator method, we used multiple imputation for variables with over 10% missing data. The amount of missing data was less than 10% per variable for lung cancer (15 year latency analysis) and colorectal cancer (no latency analysis). Most breast cancer covariates (20 year latency analysis) had less than 10% missing data. Exceptions were family history of breast cancer (10%), personal history of benign breast cancer (10%), history of oral contraceptive use (11%), history of radiation exposure (13%), menopausal hormone treatment (19%) and past use of DES (20%). For breast cancer (20 year latency), we imputed six complete data sets, and then ran the GAM model and statistics on each. We combined the six maps by pointwise averaging of odds ratios prior to exponentiation.

### Residential History

Our initial, no-latency analyses included all eligible residences, i.e., exposures occurring up to diagnosis were assumed to contribute to the risk of disease. However, cancers initiated by exposure to environmental carcinogens typically take more than a decade to develop. We therefore performed a fifteen-year latency analysis by restricting inclusion to the residences occupied by participants at least fifteen years prior to the diagnosis or index year (Residences within the fifteen year window were excluded because geographical location within that window was assumed not relevant to outcome). Between 46% and 48% of residences remained depending on the outcome. In addition, because breast cancer cases were obtained over a ten-year period, there were sufficient cases to perform a twenty-year latency analysis; 37% of residences remained eligible.

Some participants lived at more than one location on Cape Cod or more than once at a single location if they moved away and later returned. To determine the effects multiple residences may have had on maps, we also performed analyses that included for each individual only the residence of longest duration that met latency assumptions. Since the resulting data set is smaller, the optimal span chosen by the AIC is often, but not always, larger. As maps can change due to different span sizes, we also analyzed the reduced data set using the optimal span of the original data.

## Results

### Breast Cancer

**Figure 3:**
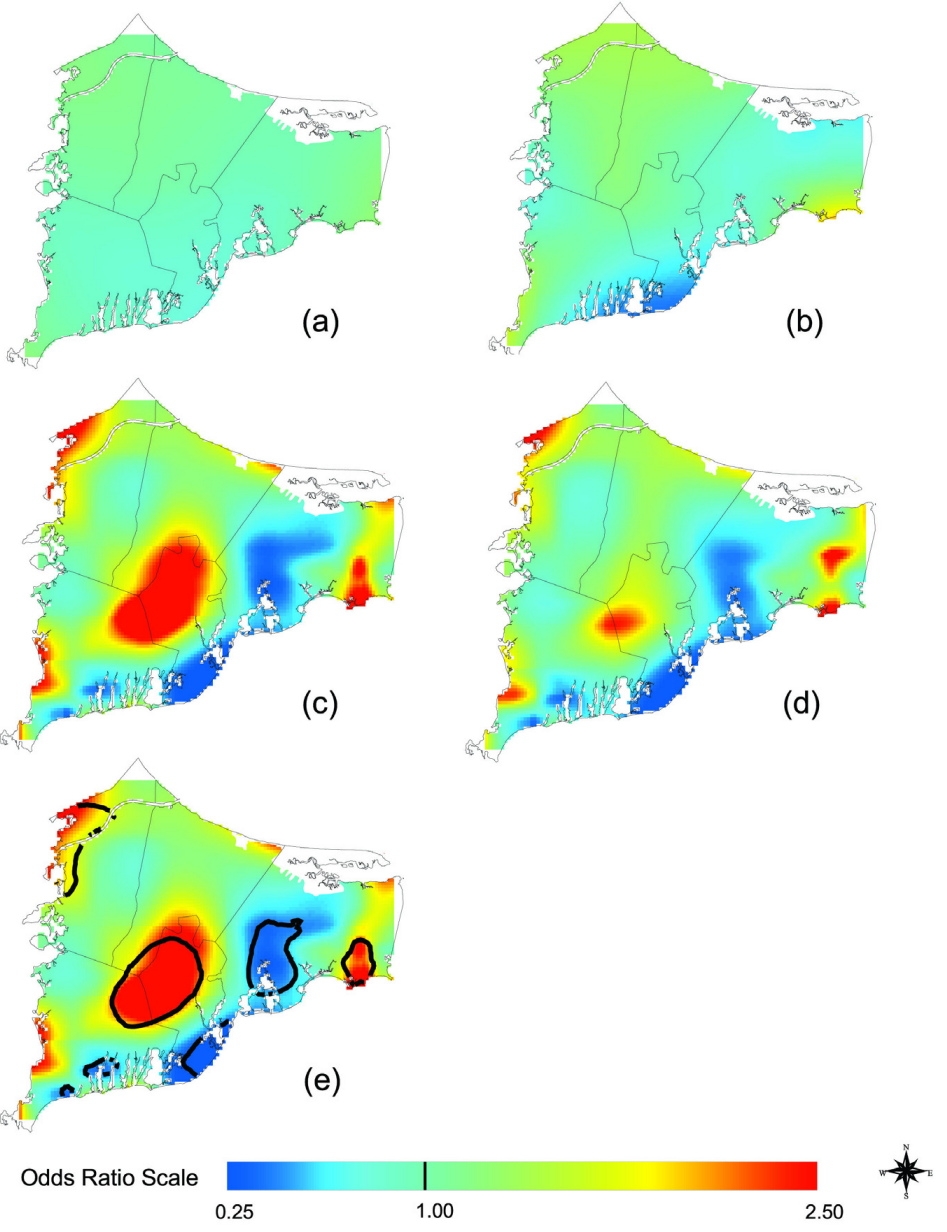
**Breast Cancer Results. **Odds ratios are relative to the whole study area. a) Adjusted, no latency. b) Adjusted. Assuming 15 years of latency somewhat increases spatial variation. c) Adjusted, 20 years of latency. Further increasing latency increases magnitude of hot and cold spots. d) Crude, 20 years of latency, created using the optimal span (0.15) of the adjusted map. Difference from the adjusted map indicates spatial confounding. e) Adjusted, 20 years of latency. Black contour lines denote areas of significantly increased and decreased risk at the 0.05 level.

Assuming no latency, location was not statistically significant at the 0.05 level (Table 1 and Figure 3a). Assuming 15 years produced a statistically significant, although still relatively flat map (Figure 3b). Assuming 20 years of latency increased the magnitude of the hot and cold spots (Figure 3c) and the overall significance of the map (Table 1). The adjusted map (Figure 3c) had more pronounced hot and cold spots than the crude map (Figure 3d). Spatial confounding was thus partially masking differences in the crude analysis. Race was the single most important variable responsible for this difference; at the time, there was a large population of Native Americans living in upper Cape Cod. The point-wise tests of significance showed a large hot spot that spans the towns of Falmouth, Mashpee, and southern Sandwich (Figure 3e). Other hot spots were identified in southeastern Barnstable and northwestern Bourne. Disease odds in certain areas were five times higher than the study area as a whole. Areas of significantly decreased risk relative to the whole study area were scattered along the southern coast of Falmouth and Mashpee and through the center of Barnstable. For individuals in the breast cancer analysis assuming 20 years of latency, 66% had only one eligible residence, 22% had two, 8% had three, and 4% had four or more.

**Table 1:** Summary of breast cancer models

Analysis	Latency (yrs)	Span^a^	Cases/ Controls	S-Plus p-value^c^	Deviance p-value^c^	Kelsall/ Diggle p-value^c^	Figure #
Adjusted All Residences	0	0.50	1061/ 1371	0.053	0.101	0.198	3a
Adjusted All Residences	15	0.35	528/ 650	0.004	0.010	0.016	3b
Crude All Residences	20	0.35	391/ 509	0.0008	0.003	0.003	---
Crude All Residences	20	0.15^b^	391/ 509	---^d^	---^d^	---^d^	3d
Adjusted All Residences	20	0.15	391/ 509	5.6E-6	0.001	0.006	3c,e 5a, 9
Adjusted Longest Duration	20	0.15^b^	248/ 341	---^d^	---^d^	---^d^	4a
Adjusted Longest Duration	20	0.45	248/ 341	0.008	0.020	0.029	4b

^a ^Optimal span obtained by using the Akaike's Information Criterion (AIC) unless otherwise noted.

^b ^Same span as for adjusted, all residences.

^c ^Null hypothesis is that the map is flat. S-Plus p-value is only approximate.

^d ^The p-values were omitted because the maps were created using a non-optimal span.

**Figure 4:**
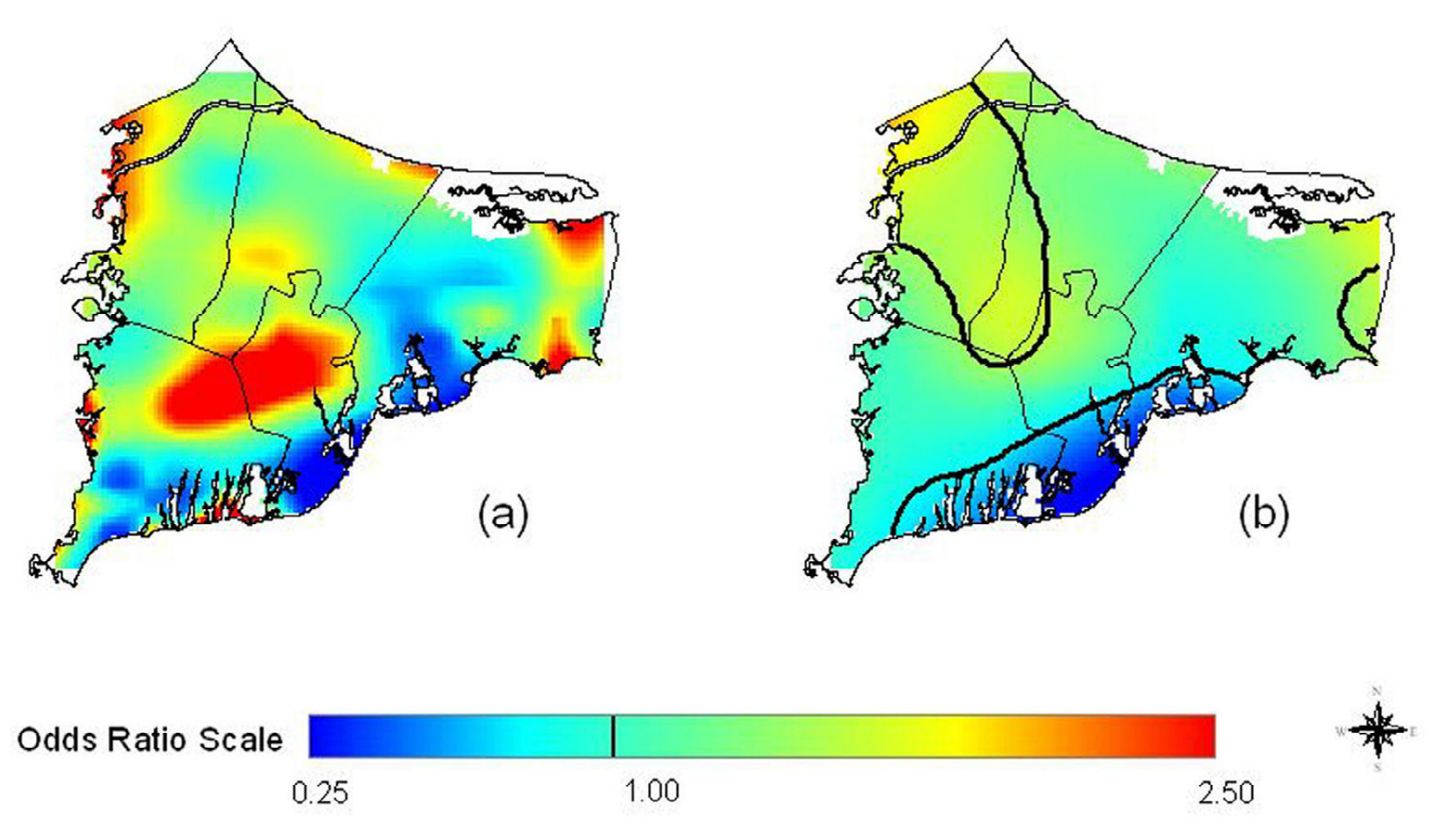
**Breast Cancer Results, Restricted to Longest Duration Residences. **a) Adjusted, 20 years of latency. Restriction to residences of longest duration has little effect when the same span (0.15) is used as for all residences (Figure 3e). b) Use of the optimal span (0.45) for the restricted analysis increases the smoothness of the map.

We next restricted the adjusted 20-year latency analysis to residences of longest duration. One third lived at their residence of longest duration for less than 20 years, 37% for 20–29 years and 30% for 30 or more years. Using the same span size as before (0.15), Figure 4a shows that although cluster size and shape has changed, the overall spatial pattern remained quite similar. The optimal span for the longest duration analysis was 0.45, and using the larger span size results in a smoother surface (Figure 4b). Hot spots and cold spots coalesced, reducing their magnitudes, but they remain statistically significant.

**Figure 5:**
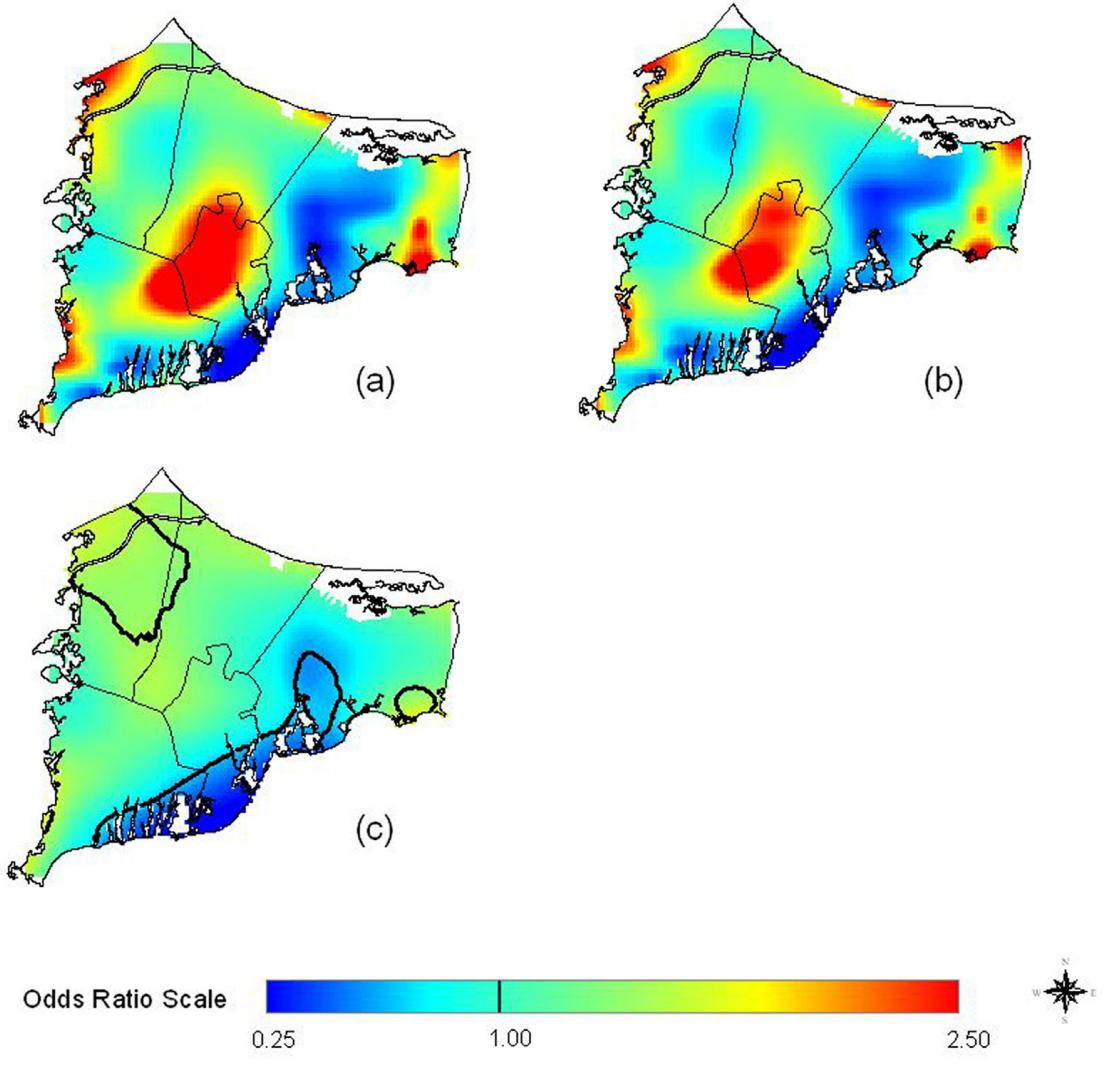
**Multiple Imputation of Missing Data had Little Effect on Breast Cancer Results. **Adjusted, 20 years of Latency. a) Breast cancer map estimated using indicator variables to signify missing covariate data (Fig. 3c). b) We imputed missing data for covariates missing 10% or more of values. We generated six data sets, applying the GAM model to each. All maps (and their average) looked virtually identical; only one is shown, drawn using the same span (0.15) as the non-imputed map in a. c) Imputed map drawn using its optimal span of 0.35. Since the span is larger, it appears smoother than in b. The global statistics for all imputed maps were highly significant, regardless of span size. Black contour lines denote areas of significantly increased and decreased risk at the 0.05 level.

**Figure 6:**
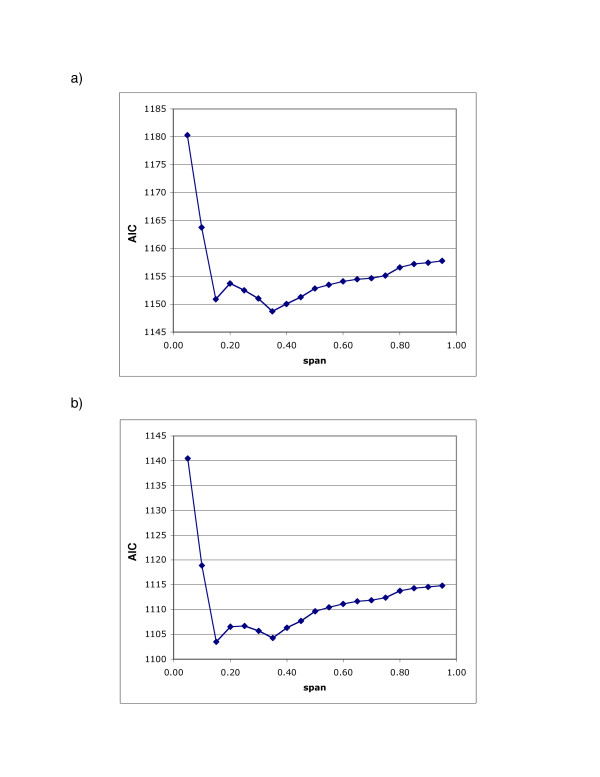
**AIC Curves for the Imputed and Non-Imputed Breast Cancer Maps. **Adjusted, 20 years of Latency. a) AIC curve for the imputed map. b) AIC curve for the non-imputed map. Both curves have local minima at span sizes of 0.15 and 0.35. Although quite similar in magnitude, the AIC value at 0.35 is slightly smaller than the value at 0.15 for the imputed map; the reverse is true for the non-imputed map. From a statistical point of view, both span sizes appear appropriate.

Compared with the original map produced using indicator variables for missing data (5a), multiple imputation had only minor effects on the appearance of the 20 year latency analysis for all residences (Figure 5b); as all six imputed maps (and their average) look virtually identical, we show only one. However, the optimal span was 0.35 for the imputed maps, larger than the span of 0.15 for the original map. At this higher span, the imputed map appears smoother (Fig. 5c). Comparison of the AIC curves for the original and imputed maps (virtually identical for the six imputed data sets) indicates that both have two local minima at spans 0.15 and 0.35 (Fig. 6a, 6b). Although quite similar in magnitude, the AIC at span 0.15 is slightly smaller for the original data set while the AIC at span 0.35 is slightly smaller for the imputed data sets. From a statistical point of view, both span sizes appear to be appropriate. However, because of the low population density around the military base, use of the larger span size tends to merge two "hot spots" in the center and the northwest corner of the map (Fig. 5b, 5c). The global statistics for the imputed maps were highly significant regardless of span size.

### Lung Cancer

**Figure 7:**
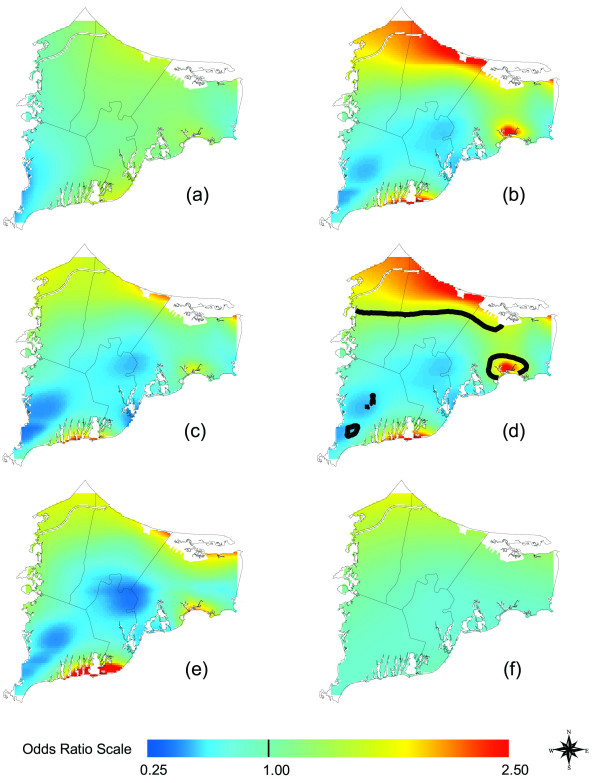
**Lung Cancer Results. **Odds ratios are relative to the whole study area. a) Adjusted, no latency. b) Adjusted, 15 years of latency. Increasing latency increases magnitude of hot and cold spots. c) Crude, 15 years of latency. Difference from the adjusted map indicates spatial confounding. The crude and adjusted maps have the same optimal span. d) Adjusted, 15 years of latency. Black contour lines denote areas of significantly increased and decreased risk at the 0.05 level. e) Adjusted, 15 years of latency. Restriction to residences of longest duration greatly changes the map compared to results for all residences even when the same span is used (0.3). f) Use of the optimal span (0.95) for the restricted analysis produces a very flat map.

Hot and cold spots became apparent as we increased latency from 0 to 15 years (Table 2 and Figures 7a, b). Adjusting for covariates increased odds ratios in the northern part of the map (Compare Figures 7b, c). Location was statistically significant for 15 years of latency. The northern region of upper Cape Cod and southern Barnstable were areas of significant increased risk relative to the entire study area, while small areas of Falmouth had areas of significant decreased risk (Figure 7d). For individuals in the lung cancer analysis assuming 15 years of latency, 61% had only one eligible residence, 22% had two, 11% had three, and 5% had four or more.

**Table 2:** Summary of lung cancer models

Analysis	Latency (yrs)	Span^a^	Cases/ Controls	S-Plus p-value^c^	Deviance p-value^c^	Kelsall/ Diggle p-value^c^	Figure #
Adjusted All Residences	0	0.95	385/ 1927	0.056	0.072	0.080	7a
Crude All Residences	15	0.30	182/ 871	0.005	0.016	0.101	7c
Adjusted All Residences	15	0.30	182/ 871	0.004	0.004	0.029	7b,d
Adjusted Longest Duration	15	0.30^b^	100/ 485	---^d^	---^d^	---^d^	7e
Adjusted Longest Duration	15	0.95	100/ 485	0.227	0.351	0.381	7f

^a ^Optimal span obtained by using the Akaike's Information Criterion (AIC) unless otherwise noted.

^b ^Same span as for all residences.

^c ^Null hypothesis is that the map is flat. S-Plus p-value is only approximate.

^d ^The p-values were omitted because the maps were created using a non-optimal span.

For the adjusted 15-year latency analysis restricted to residences of longest duration, 23% of the subjects lived at their residence for less than 20 years, 18% for 20–29 years and 30% for 30 or more years. Restricting the 15-year latency analysis to residences of longest duration changed the map, eliminating the significant hot spots and the global significance of location (Figure 7e, drawn using the same span as Figure 7d). This result may imply that inclusion of multiple residences biased the non-restricted analysis. The optimum span for the longest duration analysis increased from 0.30 to 0.95, producing a map rather flat in appearance (Figure 7f). The increased span size may be due in part to the decreased amount of data.

### Colorectal Cancer

**Figure 8:**
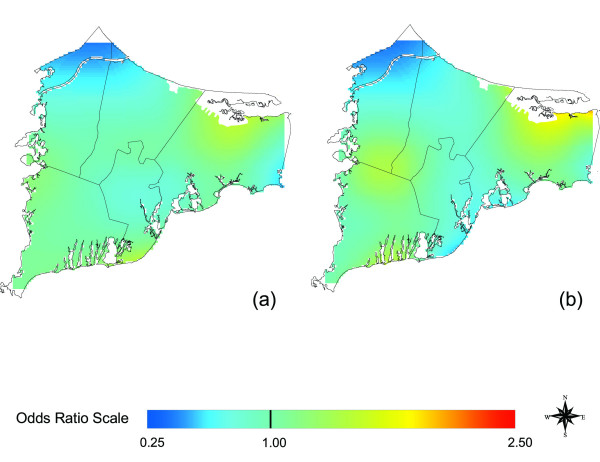
**Colorectal Cancer Results. **Increasing latency from 0 years (a) to 15 years (b) shows little effect on adjusted odds ratios. Odds ratios are relative to the whole study area.

The maps for colorectal cancer showed less variation in odds ratios than those for breast and lung cancer. Location was not statistically significant except at the less plausible assumption of no latency (Table 3). Little change was seen in the odds ratios when latency was increased from 0 to 15 years (Figure 8). Neither adjusting for covariates nor restricting to residences of longest duration had much effect on the 15-year latency analysis (maps not shown).

**Table 3:** Summary of colorectal cancer models.

Analysis	Latency (yrs)	Span^a^	Cases/ Controls	S-Plus p-value^c^	Deviance p-value^c^	Kelsall/ Diggle p-value^c^	Figure #
Adjusted All Residences	0	0.35	469/ 1791	0.002	0.006	0.013	8a
Crude All Residences	15	0.60	203/ 854	0.090	0.162	0.116	---
Adjusted All Residences	15	0.60	203/ 854	0.067	0.120	0.157	8b
Adjusted Longest Duration	15	0.60^b^	112/ 488	---^d^	---^d^	---^d^	---
Adjusted Longest Duration	15	0.95	112/ 488	0.239	0.360	0.158	---

^a ^Optimal span obtained by using the Akaike's Information Criterion (AIC) unless otherwise noted.

^b ^Same span as for all residences.

^c ^Null hypothesis is that the map is flat. S-Plus p-value is only approximate.

^d ^The p-values were omitted because the maps were created using a non-optimal span.

### Comparison of Global Statistics

As shown in Tables 1, 2, 3, the p-values computed for the permutation-based deviance test and the Kelsall-Diggle statistic were typically similar, with the former usually slightly smaller. The p-value provided by S-Plus for the deviance statistic using a chi square assumption was smaller, sometimes much smaller, than the permutation-based deviance test. While the chi square approximation provides a rough approximation, we recommend use of the permutation-based approach.

## Discussion

In our analyses, the maps of lung and breast cancer on upper Cape Cod displayed more variation when we controlled for covariates and increased latency. Location also became a statistically more important part of the model. Rather than causing disease clusters as is often assumed, spatial confounding was partially hiding areas of increased risk. If population movement is random with respect to disease status, ignoring latency should cause nondifferential exposure misclassification and tend to make maps flatter. Areas of increased and decreased breast cancer risk became more pronounced, and the maps became more statistically significant, when we increased latency from 0 to 15 to 20 years. The trend towards greater spatial variation in both breast and lung cancer with increased latency is consistent with misclassification of geographically associated risk factors, including environmental exposures. Alternatively, people who lived on the Cape for many years may have personal risk factors that we did not control. A recent non-spatial analysis also found that breast cancer risk was associated with long residence on Cape Cod [13]. In contrast, the maps of colorectal cancer were relatively flat.

A number of epidemiology studies have examined cancer and environmental exposures on Cape Cod [5-15]. Brody et al. [14] recently reported no association between breast cancer and wide-area application of pesticides, assessed using historical records and GIS. Modest increased risks were associated with aerial application of persistent pesticides to cranberry bogs and use of less persistent pesticides for agriculture and tree pests. Previous studies investigated the association between breast, lung and colorectal cancer and tetrachloroethylene in drinking water from vinyl-lined asbestos cement distribution pipes [10-12]. Moderately increased risks were found for breast and lung cancer in the most exposed individuals. In our analysis of breast cancer with twenty years of latency, 12 out of 900 residences were exposed to tetrachloroethylene from pipes, only one within a significant hot spot. For lung cancer assuming 15 years of latency, 8 out of 1053 residences were exposed, only two within a significant hot spot. Adding tetrachloroethylene to the models had no effect on the appearance of either map.

Our analysis located a significant lung cancer "hot spot" north of the Massachusetts Military Reservation (Compare Figures 1 and 7d). Earlier research had found a modest increased risk of lung cancer within 3 km of gun and mortar training sites on the military base [7]. We also found a significant breast cancer hot spot on the southeastern edge of the MMR. French and Wand [15] reported an area of increased risk for prostate cancer southeast of the MMR. Others found suggestions of a link between low birth weight and proximity to the base [27].

**Figure 9:**
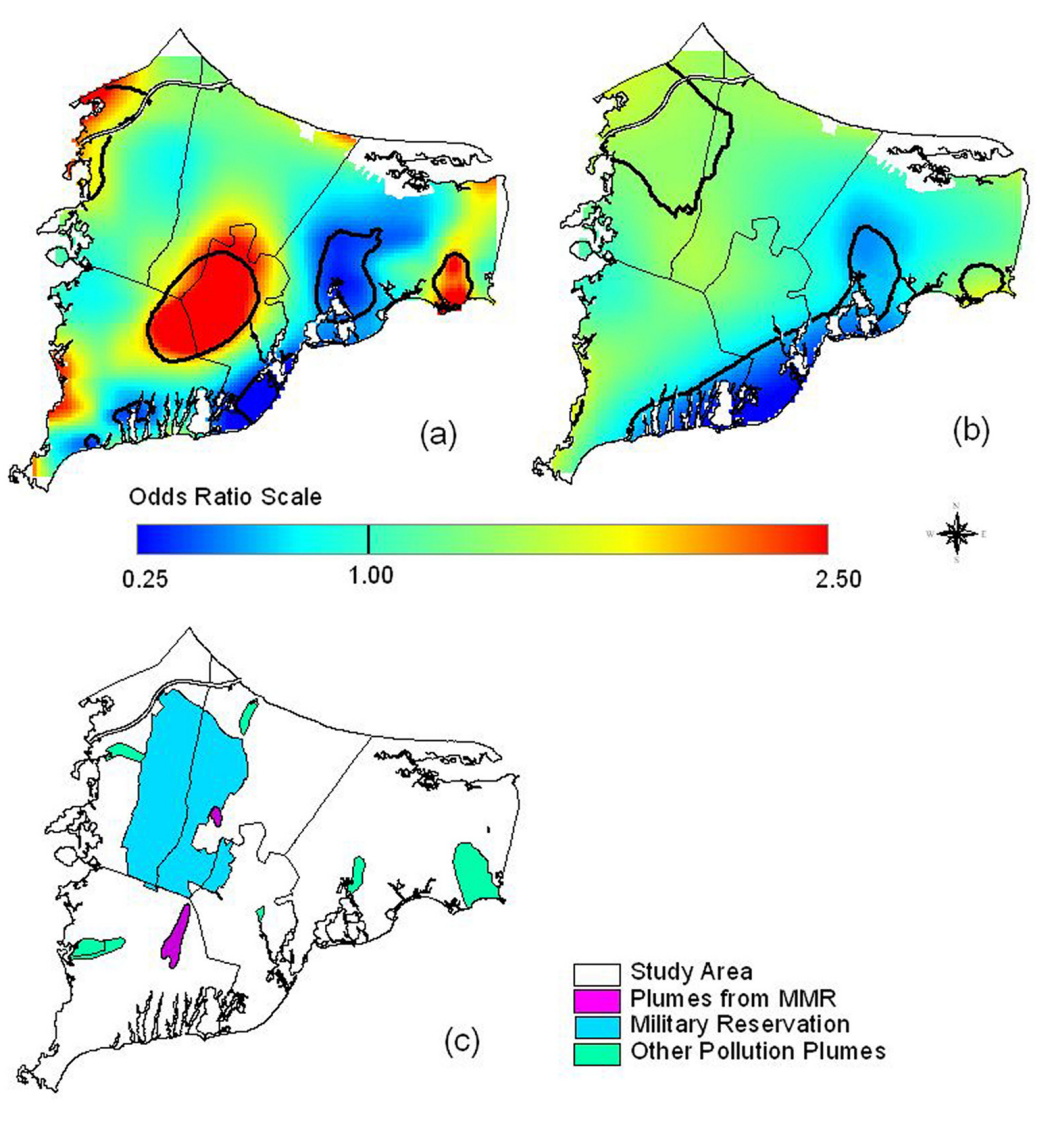
**Groundwater plumes, the Massachusetts Military Reservation (MMR), and significant breast cancer hot spots. **Adjusted, 20 years of Latency. Odds ratios are relative to the whole study area. a) Breast cancer map estimated using indicator variables to signify missing covariate data with an optimal span of 0.15 (Fig. 3c). b) Imputed map drawn using its optimal span of 0.35 (Fig. 5c). c) Location of the MMR and groundwater plumes from the MMR and other sources such as landfills. From a statistical point of view, both span sizes appear to be appropriate. However, because of the low population density around the military base, use of the larger span size tends to merge two "hot spots" in the center and the northwest corner of the map.

Overlaying maps of odds ratios with maps of pollution sources can generate hypotheses about exposure. Caution is needed, however, because many geographic features may overlap. To generate hypotheses for further investigation, we looked in a Massachusetts online repository of geographically coded features for shape files potentially related to environmental exposure [38]. Groundwater plumes were of particular interest because of earlier hypotheses that breast cancer might be related to pollution of drinking water. With no prior knowledge of any geographic relationship to breast cancer, we compared the two data sets (Figure 9), and found a suggestive overlap between the three significant breast cancer hot spots and ground water plumes, some from the MMR. Since the plumes likely did not have the same positions during the exposure period (assuming latency) and subjects variously used private wells or public water, this concordance does not establish exposure. However, this hypothesis could be tested by identifying participants' drinking water sources and comparing years of residency to the years of possible contamination.

Case-control studies are one of the standard epidemiologic tools for investigating associations between disease and exposure. By combining such data with advanced statistical techniques, we were able to address many criticisms of spatial studies. Relatively large numbers of cancer cases were ascertained from a registry and cancer types were studied separately. Point-based data from a region were used, avoiding aggregation within arbitrary political boundaries. Controls provided an estimate of the underlying, non-uniform population density. We were able to control for many covariates not available in studies that rely on registry data alone. Residential history information allowed us to take latency into account, potentially quite important for diseases like cancer.

Nevertheless, our results have a number of potential limitations. Residential locations do not account for daily movement of individuals. For breast cancer, there is a possibility that areas of elevated disease risk are due to screening bias: Women in the underlying population may have had greater opportunity for screening in these areas. We therefore examined the association between location and whether controls had undergone mammography, adjusting for age and family history of breast cancer (Mammography data were only available for the non-proxy controls). The resulting map was relatively flat and different in appearance from the breast cancer maps, suggesting no spatial screening bias (map not shown, p-value for global test = 0.18). Our use of residential history allowed us to take latency into account but produced multiple residences, a potential source of bias. Since we analyzed residences, an apparent cluster may actually be caused by a few people moving within a small area. To examine the effect of multiple residences, we restricted our analyses to residences of longest duration. Although the spatial pattern of risk was similar for breast cancer, there were differences in the location and magnitude of hot and cold spots in the lung cancer analysis. This may indicate that the inclusion of multiple residences biased the lung cancer analyses. Improved methods for analyzing data with multiple residences are needed; weighting by residence time has been suggested [39]. While missing covariate data are a potential source of bias, multiple imputation suggested little effect on the results for breast cancer with 20 years of latency. Spatial methods for analyzing data with missing covariates are underdeveloped; the recent paper by French and Wand offers another possible option [15]. We adjusted for many individual level risk factors, but some authors argue for the inclusion of group-level contextual variables, e.g. [40]. By linking residential location to census data, one could test the importance of these variables relative to individual-level covariates. No information was available on some individual-level risk factors, e.g., genetic predispositions. While areas of increased or decreased risk may theoretically be caused by non-uniform control selection, sampling of controls within the study area did not depend on geography. We computed global and pointwise p-values, but many epidemiologists prefer confidence intervals when evaluating the precision of point estimates [41]. It should be possible to compute variance bands (also known as confidence bands) for our maps [17]. We identified areas with significantly increased or decreased risk using pointwise hypothesis tests. By making these multiple comparisons we increase the likelihood of finding significant hot or cold spots by chance alone. Although we make no adjustment for multiplicity, we only conducted pointwise tests if the global deviance test indicated that the map was unlikely to be flat. The location of significant hot and cold spots should be considered as exploratory.

Since several areas of elevated risk are near the coast, edge effects must be considered; GAMs may exhibit biased behavior at the edges of the data. However, loess may be less susceptible to this problem than many smoothers [17] and preliminary work with synthetic data found little bias on edges analyzed using our method [Webster et al. submitted].

Semiparametric studies of air pollution commonly employ GAMs. The effect of interest is modeled parametrically and several covariates are modeled with smoothers. Dominici et al. [42,43] reported that S-Plus may produce a biased parametric regression coefficient with inflated standard error. Ramsay et al. [44,45] warned that stricter convergence criteria alone are not sufficient for eliminating these issues: concurvity, a nonparametric counterpart to multicollinearity, is also responsible. We used our semiparametric model differently, modeling "exposure" (location) with a smoother and covariates parametrically, and statistically testing spatial variation with permutation methods. Inflation of software-provided standard errors is thus not an issue, but bias of the smooth is not ruled out. As an initial check, we modeled synthetic data using both default and more stringent convergence parameters; the maps were very similar and simple covariates were adequately controlled [Webster et al. submitted]. Additional work is needed on this issue.

Choice of bandwidth is one of the most important issues in smoothing [17]. We used the Akaike Information Criterion, a computationally feasible method for choosing an "optimal" bandwidth based on the tradeoff between bias and variance of the smooth. There are, however, problems with automatic bandwidth selection procedures. Selecting the span that optimizes the bias-variance tradeoff is not necessarily the same as understanding the importance of map features. The optimal span tends to be larger for smaller data sets, resulting in a smoother surface. Thus certain features in the data may not be captured in the analysis (e.g., compare Figures 4a, b). Furthermore, the AIC curves for breast cancer suggest two reasonable choices of bandwidths (Figures 6a, 6b). Rather than using a single bandwidth, there may be important aspects of the data at different scales. New methods are needed to address this issue, e.g., [46].

Statistical methods for mapping adjusted, point-based epidemiologic data are still fairly novel. It would be useful in the future to compare the results of generalized additive models and generalized linear mixed models.

## Conclusion

Using generalized additive models and GIS, we generated maps of breast, lung and colorectal cancer risk. Our analyses showed little or no association between geographical location and colorectal cancer on upper Cape Cod. We observed an area of significantly elevated lung cancer risk north of the Massachusetts Military Reservation, similar to earlier research linking lung cancer to proximity to the military base. However, this result did not hold when we restricted analysis to residences of longest duration. Our results provide evidence for spatial clustering of breast cancer on upper Cape Cod. Areas of increased and decreased risk of breast cancer were not explained by covariates and became more extreme as we increased latency, findings consistent with geographical exposures. We identified three significant hot spots of breast cancer that coincide with groundwater plumes, an exposure hypothesis that warrants further investigation. We showed that spatial confounding can occur in maps, but in our analyses it tended to obscure rather than create clusters. Spatial epidemiology of population-based case-control studies addresses many methodological criticisms of cluster studies and generates new exposure hypotheses. Generalized additive models provide a relatively straightforward way to perform such analyses using standard software.

## Abbreviations

AIC, Akaike's Information Criterion

DES, diethylstilbestrol

GAM, generalized additive model

GIS, geographical information systems

MMR, Massachusetts Military Reservation

OR, odds ratio

## Competing Interests

The author(s) declare that they have no competing interests.

## Authors' Contributions

VV conducted the spatial analyses and drafted the manuscript. TW directed the study, collaborated on all analytical decisions and wrote the second draft. JW provided statistical support and consulted on analytical and editorial issues. AA provided the data and assisted in epidemiologic analysis and editing. DO participated in the design of the study and the editing of the manuscript. The first two authors contributed equally. All authors read and approved the final manuscript.
